# The 30-year incremental pattern of breast cancer: A study in the northern of Iran

**DOI:** 10.22088/cjim.14.4.720

**Published:** 2023

**Authors:** Mahboobeh Asgharian, Dariush Moslemi, Mohammad-Ali Jahani, Ali Bijani, Hossein-Ali Nikbakht, Hakimeh Mehdizadeh

**Affiliations:** 1 Student Research Committee, Babol University of Medical Sciences, Babol, Iran; 2 Department of Radiation Oncology, Babol University of Medical Sciences, Babol, Iran; 3 Social Determinants of Health Research Center, Health Research Institute, Babol University of Medical Sciences, Babol, Iran; 4 Babol University of Medical Sciences, Babol, Iran

**Keywords:** Breast cancer, Risk factor, Prevention, Metastasis

## Abstract

**Background:**

Rapid economic progress and cultural-social changes have led to lifestyle changes and increased risk of breast cancer all around the world, including Iran. This study aims to investigate the 30-year incremental pattern of breast cancer in patients of Shahid Rajaei Radiation Therapy Center in Babolsar, North of Iran.‌‌

**Methods:**

In this cross-sectional study, the data were retrospectively extracted from the physical and electronic files of patients diagnosed with breast cancer from 1992 to 2021 every 5 year by census method, during the study, overall, 1326 patients' information out of 6199 patients was analyzed using SPSSV.22 software at the level of p-value ≤ 0.05.

**Results:**

The average age of patients was 49.84 ± 11.26 years, which has been increasing over the years of study. 6143(99.13%) patients were women, the mean and standard deviation of their BMI was 29.63 ± 6.00, the number of patients with stage 1 is increasing, and patients with stage 3 is decreasing. 871(65.70%) people went through Radical Mastectomy (MRM), 261(19.68%) people experienced metastasis. There was a statistically significant relation between the type of surgery, stage of cancer, metastasis, and local recurrence within the years of study (p<0.001).

**Conclusion:**

Breast cancer and the age of getting it have increased in recent years. However, advanced stages as well as metastasis and local recurrence have decreased during the investigated years. Therefore, it is recommended to continuously warn women about the risk factors and develop suitable disease screening programs and implement them effectively.

Breast cancer is the most common malignancy that is globally diagnosed, and the leading cause of cancer-related death in women, with over 2 million deaths per year. In 2020, 685,000 deaths were reported caused by this disease (1, 2). The average rate of getting breast cancer is different in every part of the world, and the possibility of getting it, is higher in developed countries (54.5 in 100,000 people) due to the rapid economic and social growth in developing countries and lifestyle changes, this rate is increasing rapidly (3). According to the latest statistics of Globocan for the year 2020, breast cancer is the most common cancer in Iran with 16,967 cases (4, 5). According to the reports of the National Cancer Registry in the last 20 years, the risk of getting breast cancer in Iran has been increasing in men and women (6). Studies indicate that the average age of breast cancer in Iran is 46.76 ± 1.19 years and lower than the world average (7). According to a study conducted to investigate the number of cancer patients’ referrals in northern Iran, most cancer patient referrals are related to digestive, breast, and respiratory organs, which indicates the high prevalence of breast cancer (8). One of the possible consequences of breast cancer after treatment is the return of the disease in the form of metastasis or local and it has been seen that the primary cause of death in breast cancer is tumor invasion and metastasis (9).

Patients with metastasis have a shorter life span compared to other patients (10). Being aware of the risk factors alone cannot predict metastasis, because some of these factors are attributed to the individual's personality or environmental characteristics. which are specific to the individual and as a correlation factor cause a connection between the occurrence of different recurrent events for the patient (11). Due to these characteristics despite the similarity of the two individuals in terms of predictive factors, one patient sooner or later, will suffer local recurrence and metastasis (12). Today, with the advancement of science and new disease screening methods, breast cancer is diagnosed in the earlier stages of the disease, however, in patients with advanced stages, the risk of metastasis to further body parts will increase as well (13). According to a large epidemiological study in Iran, 65.5%-70.5% of patients are diagnosed in the early stages (stage 1 or 2) and less than 30% in advanced stages (14). Today, in the early stages of the disease, breast-conserving surgery (BCS) is preferred over modified radical mastectomy (MRM) (15, 16). In addition, patients have shown more satisfaction regarding psychological and beauty aspects (17).

Several studies have been conducted about breast cancer, including the following studies, the study by Bakhtiari et al., states that breast cancer is the most common type of cancer among women in the North of Iran, with a rate of 9.24% (18). The study of Enayat Rad et al., on the topic of examining the prosses of breast cancer’s changes of Iranian women in 2016 in the city of Tehran, which indicates the increase of breast cancer in population of women in the country (19). A study conducted by Lima et al., in Colombia with the aim of the global emergence of breast cancer and mortality rates, based on region, age, and fertility patterns indicated that breast cancer has an increasing pattern in all age groups and the rate of breast cancer mortality has increased by 0.23% per year, which was significant in age groups under 50 years and 70 years and older (20). In a study conducted by Ibrahim, N. et al., aimed to investigate the effect of obesity on breast cancer in 2021 in Egypt, 63.29% of patients were obese and severely obese (BMI>30) (21). The study of Steponaviciene et al., on the topic of the rate of breast cancer and the distribution of the stage before and after the introduction of the disease screening program in 2019 in Lithuania showed that the rate of localized cancer increased during the years of 2012-1998, but the rate of its advanced stages decreased and stage 1 disease increased by 10.3% per year (22). The result of Christiansen et al.'s study compared breast-conserving-surgery versus mastectomy in Denmark in 2018 and indicated that the survival of patients who underwent BCS was better compared to those who underwent mastectomy (23).

With the increase in life expectancy and aging index in the population of Iran, it is expected that the rate of various cancers to increase in the coming years and a lot of human and financial capital will be spent on diagnosis and treatment of the disease, on the other hand, in the reports related to cancer registration In Iran, less attention has been paid to the increasing pattern and its incidence. Therefore, it is necessary to investigate the incidence of breast cancer in different regions of the country, and considering the high prevalence of breast cancer in society, as well as the problems and limitations that this disease may cause for patients and the healthcare system, we decided to have a look on the 30-year pattern of breast cancer in patients of the Shahid Rajaei Radiation Therapy Center in Babolsar between the years of 1992 and 2021.

## Methods

The current study was a retrospective cross-sectional study on all breast cancer patients of Shahid Rajaei Radiation Therapy Center in Babolsar between the years 1992 and 2021. The research population included breast cancer patients who referred to Shahid Rajaei Radiation Therapy Center in Babolsar during the years 1992 to 2021, and in this regard, the total sample size was 6199 cases, and 2 cases were excluded from the study due to file defects. Except for the two variables of the year of referral and patients’ gender, which were completely extracted out of 6197 patients, for the other variables, the studied years were determined by systematic random sampling with a multiple of 5 (years 2001-2006-2006-2011-1996 2016-2021) then, using the census sampling method, relevant information was extracted from 1326 files in studied years. For data collection, a checklist was prepared including demographic information such as age, height, and weight of the patient, number of visits, type of surgery, stage of the disease, metastasis, and local recurrence using the electronic file (in cases which earlier information was not recorded, the document archive was used) and the hospital information system was extracted. Descriptive characteristics of patients were reported with statistical indicators for quantitative variables with the default of normality using the central indicators of the mean (standard deviation), median (interquartile range), minimum, max and for qualitative variables, frequency (percentage) was reported, to check the related statistical tests, the normality of the data was checked using the Kolmogorov-Smirnov test. To test the statistically significant relation between the qualitative variables, chi-square test was used and in case of limitations in the expected frequency using Fisher's exact test, also to analyze the relation between the ordinal qualitative verticals and its rate in the studied years, Two-way chi-square was used. All analyses were done by SPSS Version 22 software and Excel 2013 software was used to draw the graphs; the significance level was considered to be p<0.05.‌‌

## Results

**Figure 1:**
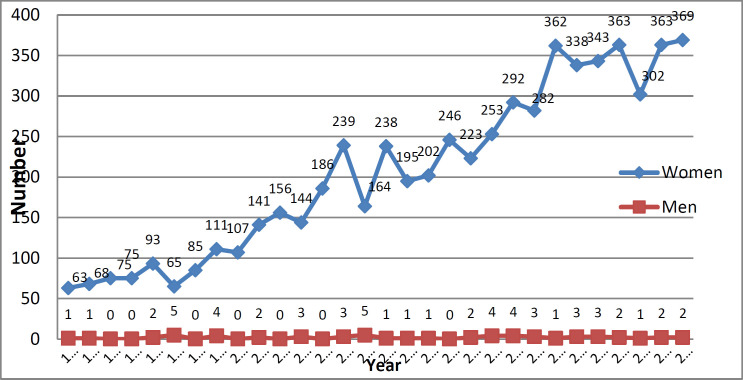
The trend of breast cancer patients referred to Shahid Rajaei Radiation Therapy Center in Babolsar by year and gender during the span of 30 years (1992-2021)

The total number of breast cancer patients during the mentioned 30-year period was 6197 cases, and the rate generally increased from 1992 to 2021, in a way that the lowest frequency was 64(1.03%) people in 1992 and the highest frequency was 367(5.92%) people in 2021. Among the 6197 patients referred to the mentioned treatment center, the most frequent 6143(99.13%) were women and 54 (0.87%) were men (figure 1).

The mean and standard deviation of the age of breast cancer patients in the studied population was 49.84 ± 11.26 years, the lowest age was 21 years and the highest was 92 years. Among the 1326 patients investigated in the target population, only 251 patients had their height and weight recorded in the file, and the mean and standard deviation of their body mass index (BMI) was 29.63 ± 6.00 (table 1).

**Table 1:** The average of some variables of breast cancer patients from 1992 to 2021

**Variable**	**Frequency**	**Mean ± SD**	**95% Confidence Intervals**	**Median (Interquartile Range)**	**Min**	**Max**
**Age**	1326	49.84 ± 11.26	49.23-50.45	49 (42-57.25)	21	92
**Frequency of Referrals**	1326	5.88 ± 8.64	5.12-6.45	2.00(1.00 -8.00)	1	87
**Body Mass Index (BMI)**	251	29.63 ± 6.00	28.94-30.44	29.21 (25.78-32.87)	15.56	51.11

**Figure 2:**
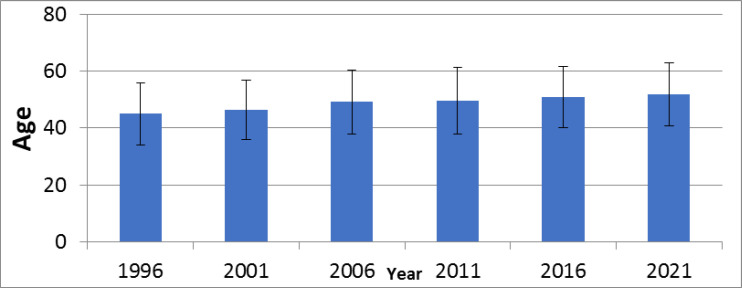
Average age and standard deviation of breast cancer patients by year from 1992 to 2021

**Figure 3:**
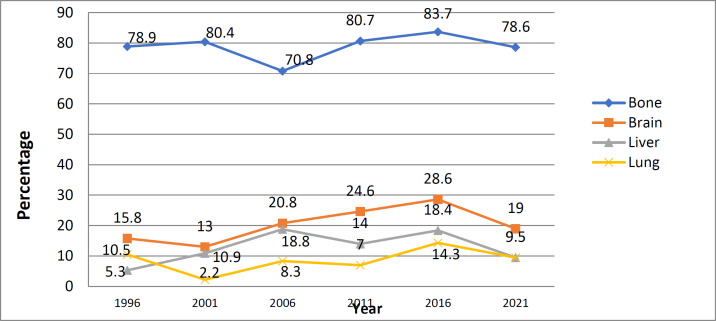
The percentage of metastatic organs of breast cancer patients separately sorted by the 6- years study (1992 to 2021)

According to figure 2, the average age of the patients has increased in the 6 years under investigation, in a way that in 1996, the average age was 44.98 years, and in 2021 it reached 51.87 years, in such a way that the difference was significant. (p<0.001). According to table 2, during the years of study, the number of patients with stage 1 has increased, and the number of patients with stage 3 has decreased. So in 1996, there were 2 (2.44%) individuals and 30(36.58%) people dealing with stage 1 and stage 3, respectively, and this number in 2021 reached 67 (19.82%) people in stage 1 and 78 (23.08%) people in stage 3. In all years, the highest frequency belonged to stage 2. Most of the patients, 871(65.70%) people went through modified radical mastectomy (MRM) surgery. Also, by examining different years, in 1996, the highest frequency of 87 (93.55%) people belonged to MRM surgery, while in 2021, the highest frequency of 173 (47.14%) people belonged to BCS surgery. Among the 1326 studied patients for this research 261(19.68%), experienced distant metastasis and 29 (2.19%) had local recurrence during the studied years. The highest metastasis was 46 (32.86%) people in 2001 and the lowest metastasis was 42 (11.44%) people in 2021. Also, the highest frequency in terms of local recurrence was 6(6.45%) people from 1996 and the lowest frequency was 2 (0.60%) individuals from 2016.It was a statistically significant relation between the stage of the disease, the type of surgery, metastasis, local recurrence, and the years of research. (p<0.001). Figure 3 shows the rate of metastases in different organs during the 6 years of the study, in every single year the highest frequency belonged to the bone, in 1996 the highest frequency was 15 (78.9%) for the bone, and the lowest frequency, 1 (5.3%) belonged to the liver and in 2021, 33 (78.6%) for the bone and 4 (9.5%) for the liver and lung at the same time. 

**Table 2:** The number of patients with breast cancer based on stage, type of surgery, metastasis, and topical recurrence separately sorted from 1992 to 2021

**status**		**Years**						**Total**
		**1996**	**2001**	**2006**	**2011**	**2016**	**2021**	
**Stage** **Frequency (%)**	**Stage1**	2(2.44)	6(4.44)	6(3.85)	28(13.08)	40(12.50)	67(19.82)	149(11. 98)
	**Stage2**	50(60.98)	82(60.74)	94(60.25)	123(57.48)	170(53.12)	185(54.73)	704(56.55)
	**Stage3**	30(36.58)	46(34.08)	50(32.05)	61(28.51)	103(32.19)	78(23.08)	368(29.57)
	**Stage4**	-	1(0.74)	6(3.85)	2(0.93)	7(2.19)	8(2.37)	24(1.90)
**P-value**		<0.001						
**Type of surgery** **Frequency (%)**	**No Surgery**	2(2.15)	2(1.43)	8(4.79)	6(2.68)	14(4.18)	27(7.36)	59(4.45)
	**Breast Conserving surgery(BCS)**	4(4.30)	12(8.57)	11(6.59)	58(25.89)	138(41.19)	173(47.14)	396(29.86)
	**Modified Radical Mastectomy(MRM)**	87(93.55)	126(90.00)	148(88.62)	160(71.43)	183(54.63)	167(45.50)	871(65.69)
**P-value**		<0.001						
**Metastasis** **Frequency (%)**	**Yes**	19(20.43)	46(32.86)	48(28.74)	57(25.45)	49(14.63)	42(11.44)	261(19.82)
	**No**	74(79.57)	94(67.14)	119(71.26)	167(74.55)	286(85.37)	325(88.56)	1056(80.18)
**Local recurrence** **Frequency (%)**	**Yes**	6(6.45)	5(3.57)	10(5.99)	3(1.34)	2(0.60)	3(0.82)	29(2.19)
	**No**	87(93.55)	135(96.43)	157(94.01)	221(98.66)	333(99.40)	364(99.18)	1297(97.81)
**P-value**		<0.001						

## Discussion

In this retrospective descriptive study, a total of 6197 patients with breast cancer were registered between 1992 and 2021, of which 6143 (99.13%) were women. The rate of getting breast cancer during the studied years shows that although there is a slight increase or decrease during these years, but generally the rate of breast cancer has been increasing in this thirty-year span. In an article published by the CDC (Centers for Disease Control and prevention) in January 2022, between the years 2004-2018, the emergence of breast cancer among women above 20 has increased by an average of 0.3% per year (24). But according to CDC statistics, studies found out that in different regions of the world annual rates of increase have been reported differently, because the emergence of breast cancer can depend on race, geographic region, and lifestyle. In a study conducted in Egypt, the annual increase rate of breast cancer emergence was reported as 2.3% (25). Therefore, the increase in the incidence of breast cancer in our region is not globally abnormal. The causes of the increase in the incidence of breast cancer could be the increase in the prevalence of obesity, drinking (alcohol), and less physical activity and lifestyle changes (24).

The average age of the patients in our study was 49.84 years. According to a systematic study of 11 Arab countries, the average age of patients was 48 years, which is almost similar to our study (26). While in the study conducted by Rachel L. Brazee et al., in America, the average age of patients was 55.3 years (27). In the study of Lukasiewiczs et al., in Switzerland, 80% of patients were over 50 years (28). Although the average age of patients in developing countries is still lower than the world average, the findings of our study have shown an increasing rate in the average age of patients during the studied years. It seems that due to the changes in the population structure and the increase in life expectancy, the age distribution of breast cancer patients has changed and the percentage of older patients has increased (29). In our study, majority of the patients have high BMI and the average BMI of the patients is 29.63. In a study conducted in Babol, most breast cancer patients were overweight (BMI: 29.9-25) (30). In the study of Lahmann et al., in Germany, it was observed that there is a direct relationship between obesity and the risk of getting breast cancer (31). As in the studies in a meta-analysis, it has been represented that every 5 unit increase in BMI is along with a two percent increase in the risk of getting breast cancer. Therefore, it is expected for the majority of patients to have high BMI score (32). In the studied years, the number of stage 1 patients was increasing and the number of stage 3 patients was decreasing, also metastasis and local recurrence in our research patients had a significant decrease annually. In a study conducted by Hirko et al., it was comprehended that for over 10 years, localized tumors and local tumors both had an annual growth in their percentage (localized tumors more than local time), and the percentage of metastatic tumors has significantly decreased during these 10 years (25). According to the studies in China and America, it has been represented that the incidence of breast cancer is higher in the early stages than in the advanced stages (33, 34).Similarly, in a study in Iran, it has also been realized that the emergence of early stages of breast cancer has increased, and this increase can be due to the increase in women's awareness of various disease screening methods, an increase in health recommendations, and an increase in the availability of mammography machines (35).

 In this study, majority of the patients went through a mastectomy, which is similar to other studies in Iran (36, 37). But it is contrary to the results of studies conducted in China and America, because, in their studies, most of the patients went through BCS surgery (33, 38). It seems that Iranian surgeons usually do not perform BCS as the first and best treatment method, one of the reasons could be the uncertainty of treatment results and the quality of existing radiotherapy services, and the possibility of patients not complying with radiotherapy (39). 

In the studied years, the most breast cancer metastasis was to the bone and the least metastasis was to the lung (except for one year in which the least metastasis belonged to the liver), while in the National Cancer Institute data review (40) the most common metastasis destinations of breast cancer, are respectively, bone (65%), lung (31%), liver (26%) and brain (9%). In other studies, almost the same results are observed in terms of the prevalence of metastasis, the most metastases are to the bone and the least are to the brain (41-43). Considering this huge difference in the results of our study and other studies in terms of the prevalence of metastases, more studies and more detailed investigations in this field are recommended. One of the limitations of this study is the defect in completing some data in the patients' files, which has led to defects in the data of this research for some variables, which has been corrected in recent years.

Generally, our study showed that being diagnosed with breast cancer and the age of the disease in our region have increased in recent years. Also, most of the patients in this study had a high BMI. Therefore, warning people about these findings and risk factors of breast cancer will have positive results. Considering the increasing rate of the initial stages of the disease at the time of diagnosis and subsequently the decreasing rate of metastasis and local recurrence in patients, the necessity of continuous use of disease screening methods, increasing women's awareness regarding the importance of self-examination by the patient and developing methods for more and more effective access of women, disease screening programs are recommended. 
